# Factors Affecting Acceptance of Life Education in Mainland China: National Cross-Sectional Study

**DOI:** 10.2196/78844

**Published:** 2026-04-21

**Authors:** Xinyue Zhang, Nana Huang, Yuyang Yi, Yinlin Wang, Wai-Kit Ming, Yibo Wu, Xiaohong Ning, Chen Wang

**Affiliations:** 1 School of Health Policy and Management, Chinese Academy of Medical Sciences & Peking Union Medical College, No. 9, Dongdansantiao Street, Dongcheng District, Beijing, 100730, China, 86-10-65120199; 2 Xiangya School of Nursing, Central South University, Changsha, China; 3 Department of Infectious Diseases and Public Health, Jockey Club College of Veterinary Medicine and Life Sciences, City University of Hong Kong, Hong Kong, China (Hong Kong); 4 Key Laboratory of Oral Biomedical Research of Zhejiang Province, Affiliated Stomatology Hospital, Zhejiang University, Hangzhou, China; 5 Department of Nursing, The Fourth Affiliated Hospital of School of Medicine, and International School of Medicine, International Institutes of Medicine, Zhejiang University, Yiwu, China; 6 Palliative Care Medicine Center, Peking Union Medical College Hospital, Beijing, China; 7 School of Population Medicine and Public Health, Chinese Academy of Medical Sciences & Peking Union Medical College, Beijing, China

**Keywords:** life education, personal intention, public education, affecting factors, cross-sectional study

## Abstract

**Background:**

In traditional Chinese culture, discussing death has always been taboo. The social environment characterized by fear, reluctance, and apprehension toward death significantly impedes the development of palliative care. Therefore, establishing a correct view of life and death and promoting life education are prerequisites for the successful implementation of palliative care.

**Objective:**

This study aimed to investigate the public acceptance of life education among individuals in China and analyze the explanatory variables.

**Methods:**

This national cross-sectional study was conducted from June 20 to August 31, 2022, encompassing 23 provinces, 5 autonomous regions, and 4 municipalities directly under the central government. A total of 21,875 participants were included. The generalized linear model was used to analyze influencing factors. Gender, major in medicine, place of residence, education level, family health, media use, etc, were analyzed as the potential variables. Acceptance scores were calculated based on a visual analog scale (VAS). Further subgroup analyses were carried out in different age and major subgroups.

**Results:**

The median (P_25_, P_75_) acceptance score for life education in the survey was 71.00 (50.00-95.00) points. Compared with females, males had lower acceptance (*β*=−2.39; 95% CI −3.08 to −1.69). Respondents who were majoring in medicine (*β*=3.13; 95% CI 1.11-5.14), residing in urban areas (*β*=1.25; 95% CI 0.46-2.04), processing a bachelor’s degree or higher (*β*=4.05; 95% CI 2.97-5.12), or having higher scores on the media use (*β*=0.49; 95% CI 0.41-0.57) had higher acceptance. Compared with people aged 12-17 years, those aged 25-44 years (*β*=−6.00; 95% CI −7.34 to −4.66), aged 45-64 years (*β*=−4.55; 95% CI −5.88 to −3.22), and 65 years or older (*β*=−2.16; 95% CI −3.78 to −0.55) had lower acceptance. For people majoring in medicine, place of residence, family health, and media use were uniquely relevant factors. Higher scores on the Perceived Social Support Scale (PSSS) and Health Literacy Scale-Short Form (HLS-SF) were also significantly associated with greater acceptance of life education.

**Conclusions:**

Gender, place of residence, education level, age, media use, perceived social support, and health literacy were identified as key factors influencing acceptance of life education, providing important evidence to inform targeted policy and educational interventions.

## Introduction

Life education is an educational process that examines the relationship between life and death and encompasses cultural and religious beliefs, as well as attitudes toward death and dying. Its purpose is to cultivate individuals’ appreciation for life and encourage individuals to reflect this orientation in everyday life [1]. In addition to an understanding of the process of death, life education also involves the study of attitudes toward death and its significance. In recent years, as life education has become more comprehensive, an increasing number of studies have demonstrated that life education reported beneficial associations in the overall health of individuals throughout the lifespan. In childhood and adolescence, it helps children and teenagers to understand themselves, their emotions, and behaviors, fostering self-esteem and confidence. Furthermore, it provides instruction on the establishment and maintenance of healthy interpersonal relationships, including those with family members, friends, and peers. It fosters respect for life, empathy, and a sense of moral responsibility [2]. In adulthood, individuals are assisted in understanding their interests, abilities, and career choices to facilitate the formulation of suitable career plans. They are furnished with the requisite knowledge and skills about the establishment of a family, reproduction, and child-rearing. Education on maintaining a healthy lifestyle, including diet, exercise, and mental well-being, is provided. In middle age, education is directed toward the assumption of familial responsibilities, including the care of children and older parents. It is recommended that individuals pursue further career development and advancement. Individuals continue to engage in personal growth and development, acquiring new skills and interests [3]. In the geriatric stage, the provision of factual knowledge about life and death enables older adults to confront death realistically, thereby reducing avoidance and encouraging autonomous end-of-life care planning. Furthermore, it enhances awareness of life quality and facilitates the formation of personal views on life and death based on individual experiences and cultural background. This enriches their later life and enhances their sense of well-being [4].

Life education is of significant importance in various aspects of human life, including individual mental health, social-cultural cognition, and health care service quality. Research conducted by Ronconi et al [5] indicates that life education suggests that life education can facilitate open discussions about life, death, and mental health. By participating in life education, individuals can enhance their comprehension of death and cultivate effective coping strategies, which may ultimately reduce fear and anxiety associated with mortality. Concurrently, Lei et al [6] discovered in their research on the educational needs of the older adults regarding death that older adults desire to express their perspectives on the passing of life through life education and mutual exchange to foster gratitude for life and acceptance of the inevitability of death. In health care settings, Lv et al [7] indicate that life education may support the preparation of professionals to care for dying patients by strengthening the competence of medical and nursing staff and promoting more respectful and compassionate attitudes toward dying and bereavement. This is in accordance with the findings of Zhang et al [8], which indicated that life education had a positive impact on the mental health of individual nurses and enhanced their ability to cope with death-related issues. Internationally, life education has been more extensively developed in several countries outside China [9]. From an early age, children are taught about the meaning, value, and dignity of life through school curricula and community activities, which helps them to develop an appropriate understanding of life and death issues [10]. In the United Kingdom, life education plays a pivotal role in health care and social work education, with institutions offering relevant courses and professional organizations such as the Life Education Association and the End-of-Life Care Association providing training and resources. In the United States, the exploration of individual views on death and societal attitudes toward life is a significant focus of university death research institutes or centers. In addition, nonprofit organizations play a role in raising public awareness of death, loss, and palliative care. In Germany, life education is primarily focused on professional training and services in the medical and psychological counseling fields, as well as the provision of life education for children and teenagers. Schools also offer courses on life and death. In Japan, life education is undergoing rapid development in schools and communities. Schools are offering life education courses, communities are promoting understanding and acceptance of death through lectures and activities, and dedicated life education institutions and projects are emerging. Taken together, these practices illustrate how life education can be implemented across the life course, supporting a more holistic understanding of life and death and potentially contributing to quality of life and social cohesion.

However, in part because of the influence of traditional culture, in China, death is frequently treated as a socially delicate topic and is often avoided in everyday conversation, a tendency shaped in part by long-standing cultural traditions. Confucian and Daoist moral frameworks—emphasizing filial obligations, ancestral continuity, and acceptance of the natural order—may contribute to restrained engagement with end-of-life issues and with services that explicitly address dying and bereavement, thereby posing barriers to the broader development of life education. At the same time, demographic aging is intensifying and reconfiguring care demands; the continued growth of the older population, alongside increasing burdens of chronic illness and functional limitations, is placing sustained pressure on health care and long-term care systems and is likely to amplify the need for support related to end-of-life understanding, communication, and planning. Within this policy environment, national initiatives such as the “Silver Economy” agenda—which prioritizes the health and well-being of older adults—create opportunities to strengthen life education as part of a broader strategy to improve quality of life across the life course. Despite these developments, life education in China has often been informed by models and practices developed in other settings, and empirical evidence remains limited regarding its cultural fit, public acceptability, and the conditions under which participation is more likely, especially among older adults. To address these gaps, the present study will examine public acceptance of life education and identify factors associated with receptivity and willingness to engage, with particular attention to older adults’ needs and issues of practical access. By clarifying who is most likely to accept life education and why, the findings are expected to inform the design and delivery of culturally responsive programs and to provide evidence relevant to policy and institutional decision-making on planning, implementation, and refinement; more broadly, the study aims to generate empirically grounded insights into feasible pathways for advancing life education in China in ways that support well-being and contribute to social cohesion.

## Methods

### Research Instrument

This cross-sectional study aims to describe the acceptance of life education by Chinese residents in an intuitive quantitative way and further reveal the relevant factors affecting their acceptance. The study uses a visual analog scale (VAS) scoring system, ranging from 0 to 100, to quantify participants’ level of acceptance of life education. Higher scores indicate a stronger willingness to accept. The fundamental objective of life education is to enhance individuals’ comprehension of life and death, instill respect for life, and encourage humane care. This form of education helps individuals to confront the inevitability of death, to overcome their fears of death, and to develop a scientific perspective on life and death. By exploring topics such as death, life education encourages individuals to reflect on the meaning of life, thereby enhancing their composure and rationality when confronted with life challenges. Furthermore, life education offers psychological support and assists individuals in establishing appropriate values to cope with various challenges encountered during the course of life with a more positive outlook (Table S1 in Multimedia Appendix 1).

The questionnaire design of this study is divided into 2 main parts. The initial section of the questionnaire is designed to collect information about the participants’ sociodemographic characteristics. This includes data on gender, age, place of residence, marital status, sibling status, income level, medical background, and the presence of chronic diseases. The second part uses a range of scale tools, including the Perceived Social Support Scale (PSSS), Family Health Scale-Short Form (FHS-SF), Health Literacy Scale-Short Form (HLS-SF), Quality of Life Scale (EQ-5D-5L), and Media Exposure Scale, to comprehensively assess the impact of participants’ social support, family health status, health literacy, quality of life, and media exposure on their acceptance and understanding of life education.

The Perceived Social Support Scale–Short Form (PSSS-SF) is a psychometric tool designed to assess perceived social support from different sources. We assessed perceived social support using the 3-item PSSS, which covers support from family, friends, and others. Each item is rated on a 7-point Likert scale (1-7), and item scores are summed to yield a total score ranging from 3 to 21, with higher scores indicating greater perceived social support. In our sample, the Cronbach α was 0.923 [11].

The FHS-SF is a multidimensional instrument designed to assess family health. A new International FHS was introduced, covering 4 dimensions, including family socioemotional health processes, family health and social style, family health resources, and external social support for the family. The scale contains a total of ten questions, which are scored using a 5-point Likert scoring method. The scale has scores ranging from 10 to 50, with higher scores indicating better family health functioning. Cronbach α coefficient was found to be 0.84 [12].

The Health Literacy Scale–Short Form (HLS-SF) is designed to assess an individual’s ability to access, process, and understand basic health information and services. We used the Chinese 12-item HLS-SF, which comprises 3 dimensions—health care, disease prevention, and health promotion—with 4 items in each dimension. Each item is rated on a 4-point Likert scale. Following the standard scoring procedure, responses are transformed to an index score ranging from 0 to 50, with higher scores indicating higher health literacy. Specifically, the mean item score was converted to a 0‐50 index using the transformation recommended in the original scale documentation. The reported Cronbach α for the Chinese version is 0.94 [13].

The EQ-5D-5L was used for this study. The EQ-5D-5L, a classic instrument comprising 5 dimensions (mobility, self-care, usual activities, pain and discomfort, and anxiety and depression), was used in this study, with a total of 5 items. The scale is scored using a Likert 5-point rating method. The index score ranges from 0 to 1, with 0 representing a person who has died and 1 representing a completely healthy person. The Cronbach α coefficient was found to be 0.857 [14].

The Media Exposure Scale is a tool designed to assess the extent to which individuals are exposed to various forms of media. The classic Media Exposure Scale, comprising 5 dimensions (seeking information, providing information, self-seeking, social interaction, and relaxation and entertainment), was used in the study. The scale comprises a total of 5 items, which are scored using a Likert 5-point rating method. The scale has scores ranging from 5 to 25, with higher scores indicating higher media exposure. The Cronbach α coefficient is 0.85 [15].

The World Health Organization-Five Well-Being Index (WHO-5) consists of a total of 5 items, covering 3 dimensions, including emotional experience, vitality level, and functional performance. It uses a 6-point Likert scoring method, with a total score ranging from 0 to 25, and the percentage score is calculated by multiplying the total score by 4. In the Chinese sample, the Cronbach α coefficient is 0.83, and both the structural validity and criterion validity are greater than 0.7 [16].

### Data Collection

The study was conducted between June 20 and August 31, 2022, encompassing 23 provinces, 5 autonomous regions, and 4 municipalities directly under the central government [17-20]. A stratified sampling technique was used to randomly select 2-6 cities in each province and autonomous region, as well as 2-6 communities in each prefecture-level city. In conclusion, a total of 148 cities were selected as survey targets. The selection was based on the population distribution characteristics obtained from the 7th National Population Census in 2021, which included data on age, gender, and urban-rural distribution [21]. The minimum required sample size was calculated using the following formula for a cross-sectional study design:


$$N=\frac{Z_{\alpha }^{2}\times p\times (1-p)}{\partial^{2}}$$

where the prevalence refers to the results of the meta-analysis by Krishnamoorthy et al [22]. The given formula establishes the probability of Type I error (α) as .05, while the acceptable error is determined to be .02. Consequently, the minimum sample size is computed as 1752 for each province, and the total sample size is 40,000. A quota sampling method was used to select community residents for the survey. In each selected city, surveyors or survey teams were openly recruited. All surveyors were required to undergo professional training, education, and a rigorous standardized training and process evaluation. The use of high-level, standardized technical terms was maintained throughout. The surveyors obtained informed consent from the interviewees before administering the questionnaire. The questionnaire number was entered into the Wen Juan Xing platform before the questionnaire was made available to the public. Trained surveyors conducted face-to-face interviews in sampled communities and entered participants’ responses into the Wen Juan Xing platform in real time (interviewer-administered data collection). Before each interview, surveyors obtained written informed consent. When technical terms appeared, surveyors provided neutral explanations to ensure comprehension, and no leading language was used. If a participant could respond verbally but could not complete the questionnaire due to physical limitations, the surveyor recorded the answers verbatim. Participants could withdraw at any time, and all data were collected anonymously.

The specific quota sampling standards are as follows: (1) age criteria: in the sample calibration stage, we applied age quota control. Based on the questionnaire’s age categories, the sample was stratified into 8 age groups, including 12‐18 years, 19‐25 years, 26‐30 years, 31‐40 years, 41‐50 years, 51‐60 years, 61‐70 years, and ≥71 years. Quota targets were defined as the age distribution of the final analytic sample (n=21,916), expressed as percentages: 14.70% (12‐18 y), 15.97% (19‐25 y), 9.75% (26‐30 y), 13.34% (31‐40 y), 16.75% (41‐50 y), 11.68% (51‐60 y), 10.54% (61‐70 y), and 7.27% (≥71 y). When the number of respondents in a given age group exceeded the target, we performed random sampling without replacement within that group to reduce the sample size to the target; when it was below or equal to the target, all respondents were retained. (2) Gender criteria: the ratio of males to females is approximately equal. (3) Urban-rural criteria: the ratio of urban to rural samples is approximately 3:2.

The study included individuals who met the following criteria: (1) participants must be at least 12 years of age. (2) They must hold Chinese nationality. (3) They must be Chinese residents who spend no more than one month abroad per year. (4) They must be willing to participate in the survey and complete the informed consent form. (5) They must be able to complete the online questionnaire independently or with the assistance of surveyors. (6) They must be able to understand the meaning of each questionnaire item. The rationale for selecting adolescents aged 12 years and older as research participants is as follows. First, it was found that as a result of improvements in educational quality and social environment, adolescents and adults have acquired knowledge about life education. Furthermore, the majority of adolescents aged 12 years and older possess the requisite conditions and environment to cultivate their distinctive perspectives on how to interact with deceased individuals. Second, it is reasonable to posit that these adolescents are sufficiently capable of understanding the questionnaire and responding through smartphones (if applicable). Based on these considerations, it is generally assumed that participants will engage with the survey objectively. Individuals with mental illnesses or other diseases, those participating in similar research projects, and those unwilling to cooperate are excluded from participation in the study.

### Statistical Methods

The data were subjected to descriptive statistical analysis using the Windows version of SPSS 26.0 (IBM Corp). Subsequently, a generalized linear model approach was used to examine the correlation between personal information (gender, region, age, and education level) and family status (marital status, children status, and siblings) with the VAS for acceptance of life education (range: 0‐100 points, higher scores indicating higher levels of acceptance; Table 1 and Figure S1 and Table S1 in Multimedia Appendix 1). Furthermore, subgroup analyses were conducted by medical background and by age group (Tables2^7^). In addition, sensitivity analyses were conducted for selected measurement instruments to evaluate the robustness of results to alternative scoring and operationalization of key scales.

**Table 1.** Multivariable generalized linear model of factors associated with life education acceptance (visual analog scale [VAS] 0‐100), Mainland China, June-August 2022.

Variate	*β*	SE	95% CI		*P* value
Gender (reference: female)					
Male	–2.39	0.35	–3.08 to –1.69		<.001
Religion (reference: no)					
Yes	–0.86	0.91	–2.65 to 0.93		.35
Medicine specialty (reference: no)					
Yes	3.13	1.03	1.11 to 5.14		.002
Place of residence (reference: rural)					
Urban	1.25	0.40	0.46 to 2.04		.002
Have debt (reference: no)					
Yes	1.54	0.38	0.80 to 2.28		<.001
Highest educational level (reference: Junior high or below)					
Senior high or specialty	0.33	0.48	–0.62 to 1.27		.50
Undergraduate or above	4.05	0.55	2.97 to 5.12		<.001
Age (years), reference: (12‐17)					
18‐24	0.35	0.75	–1.13 to 1.83		.64
25‐44	–6.00	0.69	–7.34 to –4.66		<.001
45‐64	–4.55	0.68	–5.88 to –3.22		<.001
≥65	–2.16	0.82	–3.78 to –0.55		.009
Region (reference: Eastern China)					
Central China	–1.98	0.43	–2.83 to –1.14		<.001
Western China	–2.57	0.43	–3.41 to –1.72		<.001
Chronic disease diagnosis (reference: no)					
Yes	1.37	0.45	0.48 to 2.25		.002
PSSS^a^	0.49	0.06	0.38 to 0.61		<.001
FHS-SF^b^	0.46	0.03	0.39 to 0.52		<.001
HLS-SF^c^	0.42	0.04	0.34 to 0.50		<.001
EQ-5D-5L^d^	0.20	0.09	0.02 to 0.37		.03
Media use	0.49	0.04	0.41 to 0.57		<.001

a PSSS: Perceived Social Support Scale.

b FHS-SF: Family Health Scale-Short Form.

c HLS-SF: Health Literacy Scale-Short Form.

d EQ-5D-5L: Quality of Life Scale.

**Table 2.** Subgroup models by medical background (medical vs nonmedical) for life education acceptance (visual analog scale [VAS] 0‐100), Mainland China, June−August 2022.

Variate	*β*	SE	95% CI		*P* value
Medicine specialty					
Gender (reference: female)					
Male	–0.82	1.79	–4.32 to 2.68		.65
Religion (reference: no)					
Yes	–16.73	11.67	–39.60 to 6.14		.15
Place of residence (reference: rural)					
Urban	5.36	1.96	1.51 to 9.21		.006
Have debt (reference: no)					
Yes	0.71	1.73	–2.68 to 4.11		.68
Highest educational level (reference: junior high or below)					
Senior high or specialty	25.89	23.68	–20.51 to 72.29		.27
Undergraduate or above	26.59	23.70	–19.86 to 73.03		.26
Age (years), reference: 12‐17					
18‐24	5.22	5.18	–4.93 to 15.37		.31
25‐44	2.12	5.92	–9.49 to 13.71		.72
45‐64	3.28	8.18	–12.76 to 19.31		.69
≥65	18.33	23.67	–28.07 to 64.73		.44
Region (reference: Eastern China)					
Central China	–2.30	2.19	–6.59 to 1.98		.29
Western China	–2.18	2.06	–6.23 to 1.86		.29
Chronic disease diagnosis (reference: no)					
Yes	1.62	2.82	–3.90 to 7.13		.57
PSSS^a^	0.48	0.30	–0.11 to 1.07		.11
FHS−SF^b^	0.36	0.16	0.05 to 0.67		.02
HLS−SF^c^	0.26	0.22	–0.17 to 0.69		.24
EQ−5D-5L^d^	0.51	0.45	–0.38 to 1.40		.26
Media use	1.28	0.24	0.81 to 1.75		<.001
Nonmedicine specialty					
Gender (reference: female)					
Male	–2.45	0.36	–3.16 to –1.75		<.001
Religion (reference: no)					
Yes	–0.77	0.92	–2.57 to 1.03		.40
Place of residence (reference: rural)					
Urban	1.10	0.41	0.29 to 1.91		.008
Have debt (reference: no)					
Yes	1.60	0.39	0.84 to 2.35		<.001
Highest educational level (reference: junior high or below)					
Senior high or specialty	0.35	0.49	–0.61 to 1.30		.48
Undergraduate or above	4.21	0.56	3.12 to 5.30		<.001
Age (years) (reference: 12‐17)					
18‐24	0.28	0.77	–1.22 to 1.78		.71
25‐44	–6.13	0.69	–7.49 to –4.77		<.001
45‐64	–4.65	0.69	–6.00 to –3.31		<.001
≥65	–2.31	0.83	–3.93 to –0.68		.006
Region (reference: Eastern China)					
Central China	–1.94	0.44	–2.80 to –1.09		<.001
Western China	–2.53	0.44	–3.40 to –1.67		<.001
Chronic disease diagnosis (reference: no)					
Yes	1.36	0.46	0.46 to 2.25		.003
PSSS	0.49	0.06	0.37 to 0.61		<.001
FHS−SF	0.46	0.03	0.39 to 0.53		<.001
HLS−SF	0.42	0.04	0.34 to 0.51		<.001
EQ−5D-5L	0.18	0.09	0.00 to 0.36		.05
Media use	0.47	0.04	0.38 to 0.55		<.001

a PSSS: Perceived Social Support Scale.

b FHS-SF: Family Health Scale-Short Form.

c HLS-SF: Health Literacy Scale-Short Form.

d EQ-5D-5L: Quality of Life Scale.

**Table 3.** Subgroup model for life education acceptance (visual analog scale [VAS] 0-100) among participants aged 12-17 years, Mainland China, June-August 2022

Variable	*β*	SE	95% CI	*P* value
Gender (reference: female)				
Male	–0.39	1.16	–2.66 to 1.88	.74
Religion (reference: no)				
Yes	4.45	3.15	–1.73 to 10.63	.16
Medicine specialty (reference: no)				
Yes	0.19	5.80	–11.17 to 11.55	.97
Place of residence (reference: Rural)				
Urban	4.08	1.24	1.66 to 6.51	.001
Have debt (reference: No)				
Yes	3.95	1.24	1.52 to 6.39	.001
Highest educational level (reference: junior high or below)				
Senior high or specialty	2.19	1.21	–0.18 to 4.56	.07
Undergraduate or above	–0.36	3.00	–6.24 to 5.51	.90
Region (reference: Eastern China)				
Central China	–3.11	1.40	–5.86 to –0.37	.03
Western China	–4.77	1.40	–7.51 to –2.04	.001
Chronic disease diagnosis (reference: No)				
Yes	–0.17	2.36	–4.79 to 4.45	.94
PSSS^a^	0.26	0.18	–0.09 to 0.60	.15
FHS-SF^b^	0.40	0.11	0.18 to 0.61	<.001
HLS-SF^c^	0.46	0.13	0.21 to 0.71	<.001
EQ-5D-5L^d^	0.13	0.27	−0.40 to 0.66	.63
Media use	0.55	0.13	0.30 to 0.80	<.001

a PSSS: Perceived Social Support Scale.

b FHS-SF: Family Health Scale-Short Form.

c HLS-SF: Health Literacy Scale-Short Form.

d EQ-5D-5L: Quality of Life Scale.

**Table 4.** Subgroup model for life education acceptance (visual analog scale [VAS] 0-100) among participants aged 18-24 years.

Variable	*β*	SE	95% CI	*P* value
Gender (reference: female)				
Male	–4.38	0.77	–5.88 to –2.87	<.001
Religion (reference: no)				
Yes	–2.90	3.39	–9.54 to 3.74	.39
Medicine specialty (reference: no)				
Yes	2.88	1.08	0.77 to 5.00	.008
Place of residence (reference: rural)				
Urban	4.99	0.86	3.30 to 6.67	<.001
Have debt (reference: no)				
Yes	1.93	0.77	0.43 to 3.44	.01
Highest educational level (reference: junior high or below)				
Senior high or specialty	8.20	3.08	2.16 to 14.24	.008
Undergraduate or above	8.84	3.07	2.82 to 14.85	.004
Region (reference: Eastern China)				
Central China	–3.45	0.92	–5.25 to –1.65	<.001
Western China	–3.97	0.92	–5.77 to –2.18	<.001
Chronic disease diagnosis (reference: no)				
Yes	3.83	1.21	1.45 to 6.21	.002
PSSS^a^	0.22	0.13	–0.03 to 0.48	.09
FHS-SF^b^	0.59	0.07	0.45 to 0.73	<.001
HLS-SF^c^	0.31	0.09	0.12 to 0.49	.001
EQ-5D-5L^d^	0.76	0.22	0.33 to 1.19	.001
Media use	0.78	0.10	0.58 to 0.98	<.001

a PSSS: Perceived Social Support Scale.

b FHS-SF: Family Health Scale-Short Form.

c HLS-SF: Health Literacy Scale-Short Form:

d EQ-5D-5L: Quality of Life Scale.

**Table 5.** Subgroup model for life education acceptance (visual analog scale [VAS] 0-100) among participants aged 25-44 years.

Variable	*β*	SE	95% CI	*P* value
Gender (reference: female)				
Male	–2.46	0.66	–3.75 to –1.17	<.001
Religion (reference: no)				
Yes	3.04	1.85	–0.59 to 6.67	.10
Medicine specialty (reference: no)				
Yes	5.01	3.42	–1.70 to 11.72	.14
Place of residence (reference: rural)				
Urban	0.06	0.81	–1.53 to 1.65	.94
Have debt (reference: no)				
Yes	1.32	0.67	0.01 to 2.62	.05
Highest educational level (reference: junior high or below)				
Senior high or specialty	0.80	0.99	–1.14 to 2.74	.42
Undergraduate or above	7.09	0.97	5.19 to 8.99	<.001
Region (reference: Eastern China)				
Central China	1.27	0.80	–0.29 to 2.83	.11
Western China	–0.80	0.82	–2.41 to 0.81	.33
Chronic disease diagnosis (reference: no)				
Yes	3.69	0.91	1.90 to 5.47	<.001
PSSS^a^	0.36	0.11	0.14 to 0.58	.001
FHS-SF^b^	0.45	0.06	0.33 to 0.57	<.001
HLS-SF^c^	0.46	0.08	0.31 to 0.61	<.001
EQ-5D-5L^d^	0.17	0.17	–0.16 to 0.51	.32
Media use	0.52	0.09	0.35 to 0.69	<.001

a PSSS: Perceived Social Support Scale.

b FHS-SF: Family Health Scale-Short Form.

c HLS-SF: Health Literacy Scale-Short Form.

d EQ-5D-5L: Quality of Life Scale.

**Table 6.** Subgroup model for life education acceptance (visual analog scale [VAS] 0-100) among participants aged 45-64 years.

Variable	*β*	SE	95% CI	*P* value
Gender (reference: female)				
Male	–2.31	0.68	–3.64 to –0.99	.001
Religion (reference: no)				
Yes	–3.32	1.57	–6.40 to –0.25	.03
Medicine specialty (reference: no)				
Yes	5.73	7.21	–8.41 to 19.86	.43
Place of residence (reference: rural)				
Urban	–1.32	0.79	–2.86 to 0.22	.09
Have debt (reference: no)				
Yes	0.37	0.74	–1.08 to 1.82	.62
Highest educational level (reference: junior high or below)				
Senior high or specialty	0.12	0.82	–1.49 to 1.72	.89
Undergraduate or above	4.50	1.00	2.54 to 6.46	<.001
Region (reference: Eastern China)				
Central China	–1.78	0.83	–3.40 to –0.16	.03
Western China	–1.08	0.82	–2.68 to 0.53	.19
Chronic disease diagnosis (reference: No)				
Yes	0.86	0.72	–0.55 to 2.26	.23
PSSS^a^	0.79	0.11	0.57 to 1.01	<.001
FHS-SF^b^	0.49	0.06	0.36 to 0.62	<.001
HLS-SF^c^	0.39	0.08	0.23 to 0.55	<.001
EQ-5D-5L^d^	–0.03	0.19	–0.40 to 0.35	.89
Media use	0.22	0.08	0.06 to 0.37	.006

a PSSS: Perceived Social Support Scale.

b FHS-SF: Family Health Scale-Short Form.

c HLS-SF: Health Literacy Scale-Short Form.

d EQ-5D-5L: Quality of Life Scale.

**Table 7.** Subgroup model for life education acceptance (visual analog scale [VAS] 0-100) among participants aged ≥65 years.

Variable	*β*	SE	95% CI	*P* value
Gender (reference: female)				
Male	–0.95	0.97	–2.86 to 0.96	.33
Religion (reference: no)				
Yes	–2.76	1.77	–6.23 to 0.71	.12
Medicine specialty (reference: no)				
Yes	12.42	25.37	–37.31 to 62.15	.62
Place of residence (reference: rural)				
Urban	0.84	1.03	–1.19 to 2.86	.42
Have debt (reference: no)				
Yes	3.03	1.37	0.34 to 5.72	.03
Highest educational level (reference: junior high or below)				
Senior high or specialty	–1.06	1.26	–3.53 to 1.41	.40
Undergraduate or above	1.36	2.04	–2.63 to 5.36	.50
Region (reference: Eastern China)				
Central China	–5.95	1.21	–8.31 to –3.58	<.001
Western China	–4.27	1.16	–6.55 to –1.99	<.001
Chronic disease diagnosis (reference: no)				
Yes	–1.44	1.05	–3.49 to 0.61	.17
PSSS^a^	0.94	0.18	0.59 to 1.29	<.001
FHS-SF^b^	0.17	0.10	–0.03 to 0.36	.10
HLS-SF^c^	0.53	0.11	0.31 to 0.75	<.001
EQ-5D-5L^d^	0.11	0.19	–0.27 to 0.49	.57
Media use	0.44	0.10	0.24 to 0.63	<.001

a PSSS: Perceived Social Support Scale.

b FHS-SF: Family Health Scale-Short Form.

c HLS-SF: Health Literacy Scale-Short Form.

d EQ-5D-5L: Quality of Life Scale.

### Ethical Considerations

This study was approved by the Ethics Review Committee of Shaanxi Health Culture Research Center (Approval No.: JKWH-2022‐02). All participants signed an electronic informed consent form before participating, which clearly stated the research objectives, procedures, and the right to withdraw without consequences and were informed again by dedicated staff before starting the questionnaire. For respondents aged 12‐18 years, their guardians signed informed consent forms and completed the questionnaire survey with the accompaniment of their guardians. To ensure the privacy and confidentiality of participants, all collected data are fully anonymized. The research dataset does not retain any identifiable personal information, and access is limited to authorized researchers only. The participants did not receive any economic compensation.

## Results

### Sociological Characteristics of the Population

**Figure 1.**
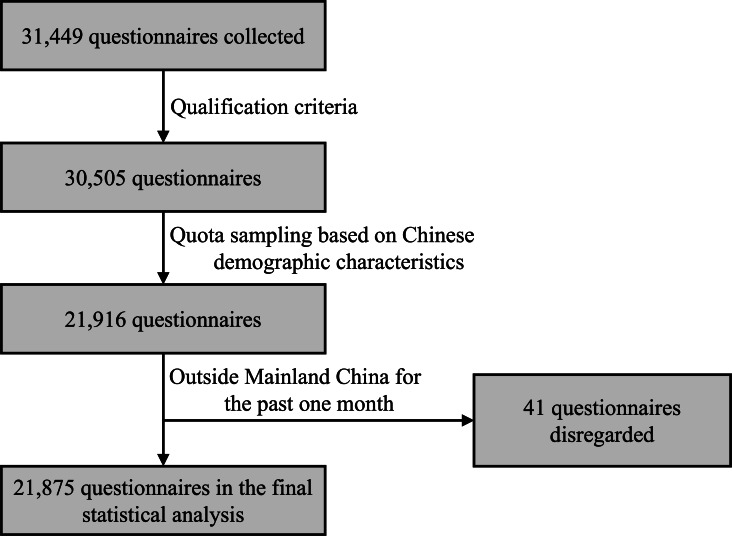
Participant low for a national cross-sectional survey of community residents in Mainland China, June-August 2022.

A total of 21,875 participants were analyzed in this study (Figure 1). Among the 21,875 respondents, 10,942 (50.2%) were female, 19,934 (91.13%) were ethnic Han, 21,005 (96.02%) were nonreligious believers, 15,155 (69.28%) resided in urban areas, and 21,137 (96.63%) were nonmedical majors. In terms of educational level, 6960 out of 21,875 (31.82%) had junior high or below, 7687 out of 21,875 (35.14%) had senior high or specialty education, and 7228 out of 21,875 (33.04%) had undergraduate or above education. Out of 21,875, a total of 8083 (36.95%) participants were from eastern China, 6905 (31.57%) participants were from central China, and 6887 (31.48%) were from western China. A total of 5652 out of 21,875 (25.84%) participants had a diagnosis of a chronic disease, and 8138 (37.20%) participants had a debt. Out of 21,875, 2170 (9.92%) participants were aged 12‐17 years, 4382 (20.03%) participants were aged 18‐24 years, 6604 (30.19%) were aged 25‐44 years, 5934 (27.13%) were aged 45‐64 years, and 2785 (12.73%) were aged 65 years or older (Table S1 in Multimedia Appendix 1).

### Acceptance of Life Education and Associated Factors

**Figure 2.**
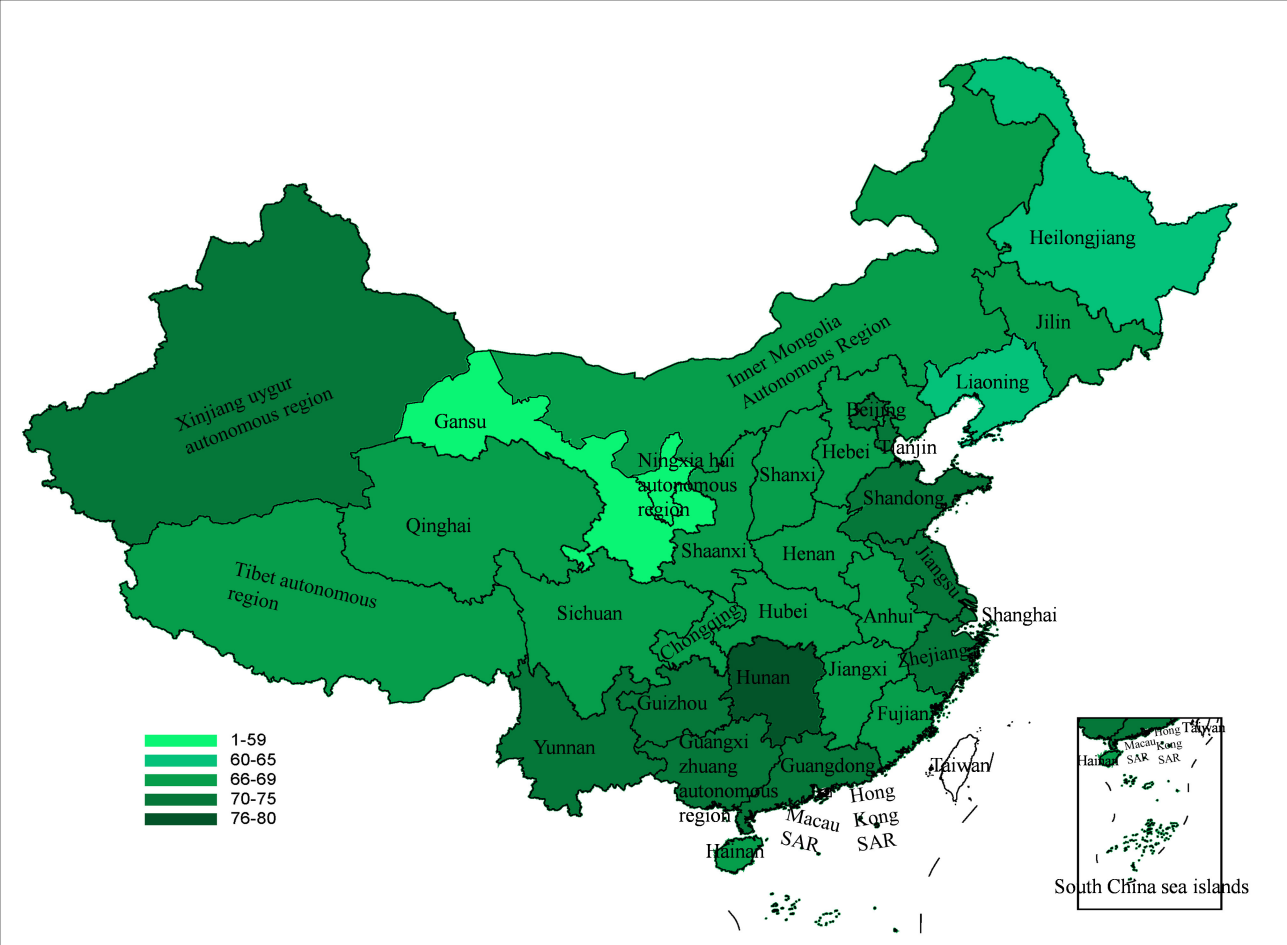
Provincial mean life education acceptance (visual analog scale [VAS] 0-100) in Mainland China, national cross-sectional survey, June-August 2022.

Among the 21,149 participants, the distribution of life education acceptance scores (VAS 0–100) was as follows: 80 (0.4%) scored 0-9; 558 (2.6%) scored 10-19; 918 (4.3%) scored 20-29; 1006 (4.8%) scored 30-39; 1802 (8.5%) scored 40-49; 2640 (12.5%) scored 50-59; 2,24 (12.9%) scored 60-69; 2347 (11.1%) scored 70-79; 2742 (13.0%) scored 80-89; and 6332 (29.9%) scored 90-100. The distribution showed a progressive increase in participant counts across higher score intervals, with the highest proportion concentrated in the 90–100 range, indicating a strong overall acceptance of life education in this population. The median (P_25_, P_75_) acceptance score for life education in this study was 71.00 (50.00-95.00), 76.7% of participants exhibited an acceptance level of 50 or above toward life education, and 6332 (28.9%) participants were listed at a 90‐100 score, which was the largest number of all people. The acceptance of life education was different in each province (Figure S2 in Multimedia Appendix 1). And the score of most provinces was more than 60, except Ningxia province and Gansu province (Figure 2). The results showed that gender, medicine specialty, place of residence, debt status, highest level of education, age, region, chronic disease diagnosis, and media use were all significant factors associated with life education acceptance (Table 1).

**Figure 3.**
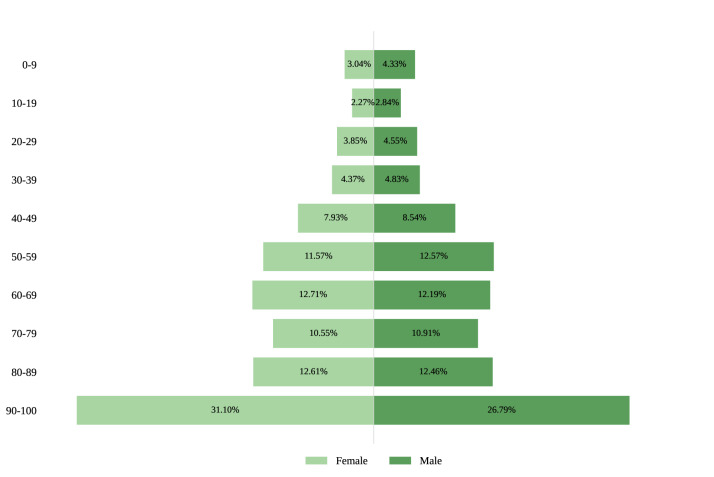
Life education acceptance (visual analog scale [VAS] 0-100) by gender in Mainland China, national cross-sectional survey, June-August 2022.

Compared with females, males had lower acceptance of life education (*β*=−2.39; 95% CI −3.08 to −1.69; Figure 3). Compared with the eastern region, the central region (*β*=−1.98; 95% CI −2.83 to −1.14) and western region (*β*=−2.57; 95% CI −3.41 to −1.72) both had lower acceptance of life education. Respondents who were majoring in medicine (*β*=3.13; 95% CI 1.11-5.14), living in an urban area (*β*=1.25; 95% CI 0.46-2.04), having debt (*β*=1.54; 95% CI 0.80-2.28), holding a bachelor’s degree or higher (*β*=4.05; 95% CI 2.97-5.12), having a chronic disease (*β*=1.37; 95% CI 0.48-2.25), or having higher scores on the Perceived Social Support Scale (*β*=0.49; 95% CI 0.38-0.61), the FHS-SF (*β*=0.46; 95% CI 0.39-0.52), the Short-Form Health Literacy Questionnaire (*β*=0.42; 95% CI 0.34-0.50), the Euro-Qol 5 Dimensions Questionnaire (*β*=0.20; 95% CI 0.02-0.37), or media use (*β*=0.49; 95% CI 0.41-0.57) had higher acceptance of life education. Compared with people aged 12-17 years, those aged 25-44 years (*β*=−6.00; 95% CI −7.34 to −4.66), aged 45-64 years (*β*=−4.55; 95% CI −5.88 to −3.22), and 65 years or older (*β*=−2.16; 95% CI −3.78 to −0.55) had lower acceptance of life education.

### Subgroup Analysis of Life Education Acceptance in Medical and Nonmedical Majors

Understanding the factors influencing the level of life education among medical professionals can guide the development of palliative care and hospice medicine, which has important practical significance. Therefore, we further analyzed medicine specialty and nonmedicine specialty subgroups (Table 2). Medicine specialty and non-medicine specialty had different acceptance levels of life education. In the medicine specialty group, only place of residence, family health (FHS-SF), and media use had statistical significance. Compared to rural areas, people from urban areas had a greater acceptance of life education (*β*=5.36; 95% CI 1.51-9.21). Higher scores on the FHS-SF (*β*=0.36; 95% CI 0.05-0.67) and media use (*β*=1.28; 95% CI 0.81-1.75) had more acceptance of life education. For the nonmedicine specialty group, most of the influencing factors were the same as the whole population.

### Subgroup Analysis of Life Education Acceptance of Different Ages

**Figure 4.**
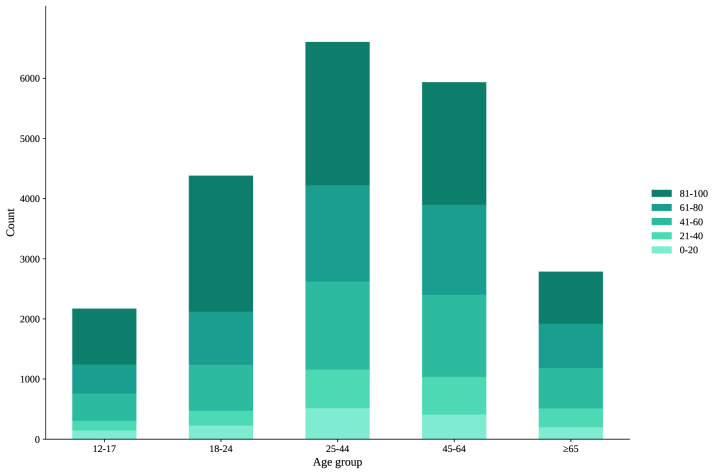
Life education acceptance (visual analog scale [VAS] 0-100) by age group in Mainland China: national cross-sectional survey, June-August 2022.

The essence of life education is the education of the whole life cycle, so age is a worthwhile variable to pay attention to (Table 3). Therefore, we further analyzed the influencing factors of different age subgroups. People of different ages had different acceptance levels of life education (Figure 4). HLS-SF and media use were the common influencing factors of all ages and were positively correlated with the acceptance of life education. Besides, those aged 12-17 years, who resided in an urban area (*β*=4.08; 95% CI 1.66-6.51), and had debt (*β*=3.95; 95% CI 1.52-6.39) had a higher acceptance of life education. The higher the score of the FHS-SF (*β*=0.40; 95% CI 0.18-0.61), the higher the acceptance level of life education they had. People aged 18-24 years had approximately the same acceptance level as all ages. However, perceived social support (PSSS) had no significance, and the higher the score of the EQ-5D-5L (*β*=0.76; 95% CI 0.33-1.19), the higher the acceptance level of life education they had, which was different across all ages. For those aged 25 -44 years, having debt (*β*=1.32; 95% CI 0.01-2.62), an educational level of undergraduate or above (*β*=7.09; 95% CI 5.19-8.99), having chronic disease (*β*=3.69; 95% CI 1.90-5.47), a higher score on the PSSS (*β*=0.36; 95% CI 0.14-0.58) and a higher score on the FHS-SF (*β*=0.45; 95% CI 0.33-0.57) exhibited higher acceptance of life education. However, medicine specialty, place of residence, and region had no significance, which was different from the findings in the overall sample. For those aged 45-64 years, those with an undergraduate degree or above (*β*=4.50; 95% CI 2.54-6.46) had higher acceptance of life education. Compared to eastern China, central China (*β*=−1.78; 95% CI −3.40 to −0.16) had lower acceptance of life education. Medicine specialty, place of residence, having debt, and chronic disease diagnosis had no significance, which was inconsistent with the results observed in the overall sample. For those aged older years, the higher score on the PSSS (*β*=0.94; 95% CI 0.59-1.29), the higher acceptance level of life education they had. Compared to eastern China, central China (*β*=−5.95; 95% CI −8.31 to −3.58), and western China (*β*=−4.27; 95% CI −6.55 to −1.99) had lower acceptance of life education. Gender, medicine specialty, place of residence, the highest level of education, chronic disease diagnosis, and family health (FHS-SF) had no significance, which were differences across all ages.

## Discussion

### Principal Findings

The definition and essence of life education should be education for all and education throughout the life cycle [23]. This study was a large-sample cross-sectional survey on the acceptance of life education and its influencing factors among Chinese residents. It is important for promoting the acceptance of life education among the general public and the development of life education.

This study found that, overall, residents had a relatively high level of acceptance of life education, and acceptance was notably high among medical professionals. However, acceptance levels were lower among older residents, males, those with lower levels of education, and rural residents. While the findings regarding religious belief were nonsignificant. Media use, gender, place of residence, education level, and age were identified as the main factors influencing the level of acceptance of life education.

The subgroup analysis in medicine indicated that place of residence, family health, and media use were factors influencing the acceptance level of life education among medical professionals. Age subgroup analyses revealed that the influences on the acceptance of life education varied across age groups. Among them, health literacy and media use were common influencing factors across age groups. Besides, the age group of 12‐17 years was also influenced by residence, debt situation, and family health. Additionally, religious beliefs were a relatively special influencing factor among residents aged 45‐64 years. For the group aged 65 years and older, the debt situation, region, and social support were the influencing factors.

Life education had lower acceptance among the older adult population, possibly due to the influence of traditional cultural beliefs. Older adult individuals may avoid discussing topics related to death, which inhibits their ability to face and accept death calmly. Consequently, they may struggle to fully comprehend the true essence of life [4,24]. Therefore, it is important to actively provide end-of-life education to the older adult population. Helping them understand that the end of life is a natural process can assist in fostering a healthy perspective on life and death. In contrast, the younger generation shows higher acceptance, primarily due to school-based curricula targeting students [21,22]. However, further engagement is still needed to cultivate early awareness of health and interpersonal relationships, fostering positive perspectives on life and death for their future. Another important objective is that young people, as children in the family, are important decision-makers for the affairs of older adults [25]. Only when the young people have a correct understanding of life and death can they help the older adults cope with death in a better way. This study also found that the acceptance of life education among medical professionals was high, reaching a score of 85, which is 14 points higher than the average and should be maintained or further improved in the future. This is because medical professionals, as a special group closely associated with life and death, undertake various tasks such as saving lives, providing life education, and offering end-of-life care [26-28]. Therefore, this group must possess a deep understanding of life and assume the responsibility of public education. Enhancing their capabilities is crucial for advancing hospice care and ensuring a meaningful life journey for patients.

In terms of gender, females are more receptive to life education than males, which is consistent with the findings of several previous studies [29-31]. Traditional Chinese gender roles influence the acceptance of life education. As primary caregivers for older adults, females are more exposed to the end-of-life process, enabling them to be better prepared for death than males, who typically focus on providing for the family [32]. Females build resilience by viewing death as a natural process and engaging in open family discussions. Conversely, males, burdened by societal expectations as primary providers, often perceive death as a heavy subject. They tend to avoid such discussions to maintain a positive, strong image for their children [33]. Therefore, females tend to have a higher acceptance of life education.

Individuals with higher media use have a higher level of acceptance of life education. Media use refers to the use of the internet, mobile phones, and television as channels for accessing desired information [34]. The widespread use of the internet provides individuals with greater access to knowledge about life education. Therefore, the higher the level of media exposure, the higher the acceptance of life education. The findings of previous studies [35,36] have consistently demonstrated that media usage can effectively enhance individuals’ access to safety and health information, foster interpersonal connections, facilitate knowledge acquisition and sharing, and serve as a valuable tool for health education. Therefore, it is a good way to carry out life education through the internet media. But since the application of internet media is a double-edged sword [37,38], caution must be exercised regarding its method and extent of application.

The findings regarding religious belief in this study were nonsignificant, deviating from prior research outcomes. Previous research [39,40] indicated that within religious contexts, death is often perceived as a natural occurrence, prompting individuals to turn to religious beliefs for solace, purpose, and inner peace. Consequently, individuals with religious convictions tended to be more receptive to life education. However, in China’s general environment, irreligious beliefs prevail [41], so the results of this study were not significant. Other studies have shown that traditional Chinese culture, influenced by Confucianism, Taoism, and Buddhism, regards life as a sacred and noble symbol, advocates caring for and cherishing life, and the majority of people show a psychology of denial, avoidance, and rejection of death, and therefore have a low degree of acceptance of life education [42]. Another interesting finding of this study was that the acceptance of life education among individuals in debt is unexpectedly high, contrary to previous research [43,44]. This may be related to the types of individuals’ debts and individuals’ perceptions of debt. For example, compared to other types of debts, student loans often cause greater stress and impact on mental and physical health [45]. However, when people do not perceive loans as a source of stress, but rather as a motivation for life’s endeavors [46], they tend to be more positive, striving to live actively, cherishing life, embracing new experiences, and thereby exhibiting a higher acceptance of life education.

The results of the medical subgroup showed that only place of residence, family health, and media use were significant, which differs from previous studies. This may be due to the smaller sample size of the medical subgroup. In previous studies [47-50], there has been a greater focus on the acceptance of life education among medical professionals. It has been found that factors such as participation in palliative care activities, level of education, and attendance at training sessions related to death all have significant impacts. There is a close relationship between better acceptance and mastery of death-related knowledge and the sustained and healthy development of palliative and hospice care. To enhance professional capacity, schools should integrate life education and palliative care into curricula, emphasizing clinical practice to deepen medical students’ understanding. Similarly, hospitals must provide continuing education to keep staff engaged in these topics. Furthermore, prioritizing cultural sensitivity, personalized care, and interdisciplinary collaboration is essential to improve patient services and advance the field of palliative medicine [51,52].

In terms of age subgroups, the influences on the acceptance of life education varied by age group, with health literacy and media use as influences common to all age groups, with higher levels being associated with higher acceptance of life education. Health literacy is defined as a cognitive and social skill that determines an individual’s motivation and ability to acquire, understand, and use information in ways that promote and maintain health [53]. Individuals with high health literacy are more receptive to health information, making them more likely to accept life education [54-56]. For aging groups 12‐17 years, the higher the level of family health, the higher the acceptance of life education [57,58]. Therefore, family-oriented life education can be conducted to enhance the acceptance level of life education among adolescent groups [59]. In addition, this study segmented the age group of young adults (18-24 years) for research because young adults are transitioning from school to society, experiencing corresponding changes in their thoughts and beliefs [60]. Therefore, life education for this age group holds significant importance. For this group, we should emphasize the dissemination of the meaning of life and knowledge about life safety. Focusing on themes such as “appreciating life,” “respecting life,” “cherishing life,” and “nurturing life,” the education should progress step by step, from shallow to deep, from theory to practice. This allows young people to appreciate the value of life and cherish it. For the group aged 65 years and above, individuals with higher levels of social support tend to have a higher acceptance level. Therefore, for the older population, we should continually strengthen social support and provide education on death-related knowledge to help them develop a correct perspective on life and death. First, it is necessary to create a societal atmosphere where discussions about death can be openly held. Building on this foundation, we can assist the older adults in understanding the concepts of life and death, recognizing the life cycle and the aging process. Gradually, as they come to understand that death is a natural part of life, we can help them initiate rational “end-of-life” planning. This involves informing them that palliative care is a scientific approach to ensuring a peaceful end, reducing fear, and helping them to face the final stages of life calmly [4].

This shift in societal development patterns and the deepening of population aging have brought to light various issues regarding life education in our country. These issues primarily manifest in the following aspects: First, this target group of life education is mainly students [29,32]. Second, most life education primarily focuses on topics related to “life,” with fewer discussions on topics related to death [61]. Third, there is a lack of specialized legal regulations and policy support specifically targeting life education (summary of existing legislation, see Table 8). The existing policies are broad in scope and lack specificity, making it difficult to directly focus on the essence of life and education. Therefore, they are unable to provide direct guidance for the promotion and development of life education. Fourth, there is insufficient interdisciplinary collaboration in the development of life education. The connections between disciplines such as education, medicine, and psychology are not sufficiently tight, making it difficult to establish comprehensive life education programs.

**Table 8.** National laws and policies related to life education in China: domains, key content, and source documents.

Category	Content of the law	Source
Educational programs and pathways	The state integrates health education into the national education system: schools use various forms to implement health education and offer health courses [62]. Encourage citizens to establish and practice the health management concept of being responsible for their own health, actively learn health knowledge, improve health literacy, and strengthen health management. Encourage family members to care for each other and form a healthy lifestyle that suits themselves and their family [62]. The state encourages the development of various forms of continuing education so that citizens can receive appropriate forms of education in politics, economics, culture, science, technology, business, etc, promotes mutual recognition and connection of different types of learning results, and promotes lifelong learning for all [63]. The state encourages social groups, social and cultural institutions, and other social organizations and individuals to carry out social and cultural education activities that are beneficial to the physical and mental health of the educated [63]. Integrating comprehensive life safety and health education into primary and secondary school curriculum and textbooks, fostering a consciousness of cherishing life and self-protection at the primary school stage, teaching objective self-awareness and self-treatment at the middle school stage, and delving into the significance and value of healthy life at the high school stage [64]. Establish and improve a health promotion and education system, improve health education service capabilities, and popularize health science knowledge from an early age. All types of media at all levels should increase the publicity of health science knowledge, actively build and standardize various radio and television and other health columns, and use new media to expand health education [65]. Increase publicity on mental health among the people and improve their mental health literacy. Strengthen intervention for common mental disorders and psychological behavioral problems such as depression and anxiety, and increase early detection and timely intervention of psychological problems among key groups [65].	Basic Medical Care and Health Promotion Law of the People’s Republic of China (December 28, 2019) Chapter 6, Article 68 Basic Medical Care and Health Promotion Law of the People’s Republic of China (December 28, 2019) Chapter 6, Article 69 Education Law of the People’s Republic of China (April 29, 2021) Chapter 2, Article 20 Education Law of the People’s Republic of China (April 29, 2021) Chapter 6, Article 53 Guidelines on Life Safety and Health Education in Primary and Secondary School Curricula and Materials (October 26, 2021) “Healthy China 2030” Plan Outline (October 25, 2016) Chapter 4, Article 1 “Healthy China 2030” Plan Outline (October 25, 2016) Chapter 5, Article 3
Legislative and policy guarantees	The state and society respect and protect the right to health of citizens: the state establishes a health education system to ensure citizens’ right to access health education [62]. Governments at all levels prioritize the health of the people in their development strategies [62]. The State Council and governments at all levels lead the work of medical and health care and health promotion [62]. The state strengthens basic scientific research, supports the development of clinical medicine, and promotes the transformation and application of medical scientific and technological achievements [62]. National health is the fundamental goal of building a Healthy China. Focusing on the entire population and across the entire lifespan, efforts are made to provide equitable and accessible, systematic and continuous health services, achieving a higher level of national health [65]. Medical and health institutions at all levels should collaborate and cooperate to provide citizens with comprehensive and continuous medical and health services throughout the entire spectrum of prevention, health care, treatment, nursing, rehabilitation, and palliative care [62].	Basic Medical Care and Health Promotion Law of the People’s Republic of China (December 28, 2019) Chapter 1, Article 4 Basic Medical Care and Health Promotion Law of the People’s Republic of China (December 28, 2019) Chapter 1, Article 6 Basic Medical Care and Health Promotion Law of the People’s Republic of China (December 28, 2019) Chapter 1, Article 7 Basic Medical Care and Health Promotion Law of the People’s Republic of China (December 28, 2019) Chapter 1, Article 8 “Healthy China 2030” Plan Outline (October 25, 2016) Chapter 2 Basic Medical Care and Health Promotion Law of the People’s Republic of China (December 28, 2019) Chapter 3, Article 36
Resource allocation	The state increases its financial investment in medical and health care and health services [62]. . The state promotes national health informatization and accelerates the construction of medical and health information infrastructure [62]. Establish a mechanism for investment in medical and health care and health services that is commensurate with economic and social development, fiscal conditions, and health indicators [62]. The state establishes a system that mainly focuses on financial allocations and is supplemented by raising education funds through various other channels [63].	Basic Medical Care and Health Promotion Law of the People’s Republic of China (December 28, 2019) Chapter 1, Article 11 Basic Medical Care and Health Promotion Law of the People’s Republic of China (December 28, 2019) Chapter 3, Article 49 Basic Medical Care and Health Promotion Law of the People’s Republic of China (December 28, 2019) Chapter 7, Article 80 Education Law of the People’s Republic of China (April 29, 2021) Chapter 7, Article 54
Training of professionals	The state develops medical education, improves the medical education system, and vigorously cultivates medical and health talents [62]. The state formulates a training plan for medical and health personnel, establishes a medical and health personnel training mechanism and a supply and demand balance mechanism that adapt to industry characteristics and social needs, and improves medical school education, post-graduation education and continuing education systems [62]. Governments at all levels should strengthen health education work and the training of health education professionals, establish systems for disseminating core information on health knowledge and skills [62]. Strengthen the coordination of medicine and education and establish and improve the supply and demand balance mechanism for medical talent training. Reform the medical education system and accelerate the establishment of a medical talent cultivation and training system that is organically connected with the three stages of institutional education, post-graduation education, and continuing education that adapts to the characteristics of the industry [65].	Basic Medical Care and Health Promotion Law of the People’s Republic of China (December 28, 2019) Chapter 1, Article 8 Basic Medical Care and Health Promotion Law of the People’s Republic of China (December 28, 2019) Chapter 4, Article 52 Basic Medical Care and Health Promotion Law of the People’s Republic of China (December 8, 2019) Chapter 6, Article 67 “Healthy China 2030” Plan Outline (October 25, 2016) Chapter 22, Article 1

Therefore, in response to these issues with life education in China and considering its influencing factors, the following improvement measures are proposed: First, tailor personalized educational programs [4,29]. Develop life education plans tailored to different groups, taking into account their cultural backgrounds, regional characteristics, and economic conditions, especially targeting young people, older adults, and male populations. Second, diverse learning pathways should be established [66,67]. Community outreach via radio, bulletin boards, and workshops can enhance awareness. Additionally, flexible online courses should be developed to complement traditional offline methods, catering to different demographics. Third, a comprehensive support system involving families, communities, and government agencies should be established [68]. This network facilitates resource sharing and fosters mutual assistance at the family and community levels. Furthermore, the government should increase comprehensive resource allocation to ensure the sustainability and expansion of life education programs. Additionally, regular policy assessments are essential to optimize their effectiveness and maximize impact. Fourth, train promoters and cultivate medical professional teams [69]. Train community workers and volunteers to become advocates and educators for life education. Disseminate health knowledge in communities to enhance the acceptance and effectiveness of life education. At the same time, it is necessary to strengthen the construction of palliative care disciplines and the professional team of palliative care professionals so as to make a good manpower reserve for hospice.

This study has several strengths. First, the large sample size reduces random sampling error and increases the precision of estimates. Second, by examining factors associated with acceptance of life education, our findings may inform the design of public education strategies and support the integration of hospice and palliative care into broader health promotion efforts. This study also has limitations. The cross-sectional design limits causal inference: the observed associations do not establish temporality (ie, whether exposures preceded acceptance), and reverse causation is possible. Although we adjusted for multiple covariates, residual confounding from unmeasured factors may remain. Future research using longitudinal designs and, where feasible, intervention-based studies is warranted to clarify causal pathways and to test whether changes in modifiable factors (eg, health literacy or media exposure) lead to changes in acceptance of life education.

### Conclusion

This study found that gender, age, media use, place of residence, level of education, perceived social support (PSSS), and health literacy (HLS-SF) are key factors influencing the acceptance of life education among Chinese residents. These findings suggest that both structural factors (sociodemographic characteristics) and modifiable psychosocial factors (social support and health literacy) should be considered when designing targeted improvement measures. Accordingly, reinforced life education interventions are particularly needed for key populations such as older adults, youth, and medical professionals. These results have important practical and contemporary value for advancing life education across the life course, improving population quality of life, and supporting the development of palliative care.

## Supplementary material

10.2196/78844 Multimedia Appendix 1 Supplementary materials from the national cross-sectional survey on life education acceptance in mainland China, including provincial variation, descriptive statistics of acceptance scores, and measurement instruments with scoring and reliability information.

10.2196/78844 Checklist 1 STROBE checklist.

## Abbreviations

EQ-5D-5L: Quality of Life Scale
FHS-SF: Family Health Scale-Short Form
HLS-SF: Health Literacy Scale-Short Form
PSSS: Perceived Social Support Scale
VAS: visual analog scale
WHO-5: World Health Organization-Five Well-Being Index
