# Supplementary material for: Factors Affecting Acceptance of Life Education in Mainland China: National Cross-Sectional Study
Source: JMIR Public Health Surveill. 2026 Apr 21;12:e78844. doi: 10.2196/78844 (PMC13100709; doi:10.2196/78844)
Supplement: Multimedia Appendix 1 [file publichealth-v12-e78844-s001.docx]

# **Figure S1. Provincial mean life education acceptance (VAS 0–100) in mainland China: national cross-sectional survey, June–August 2022.**


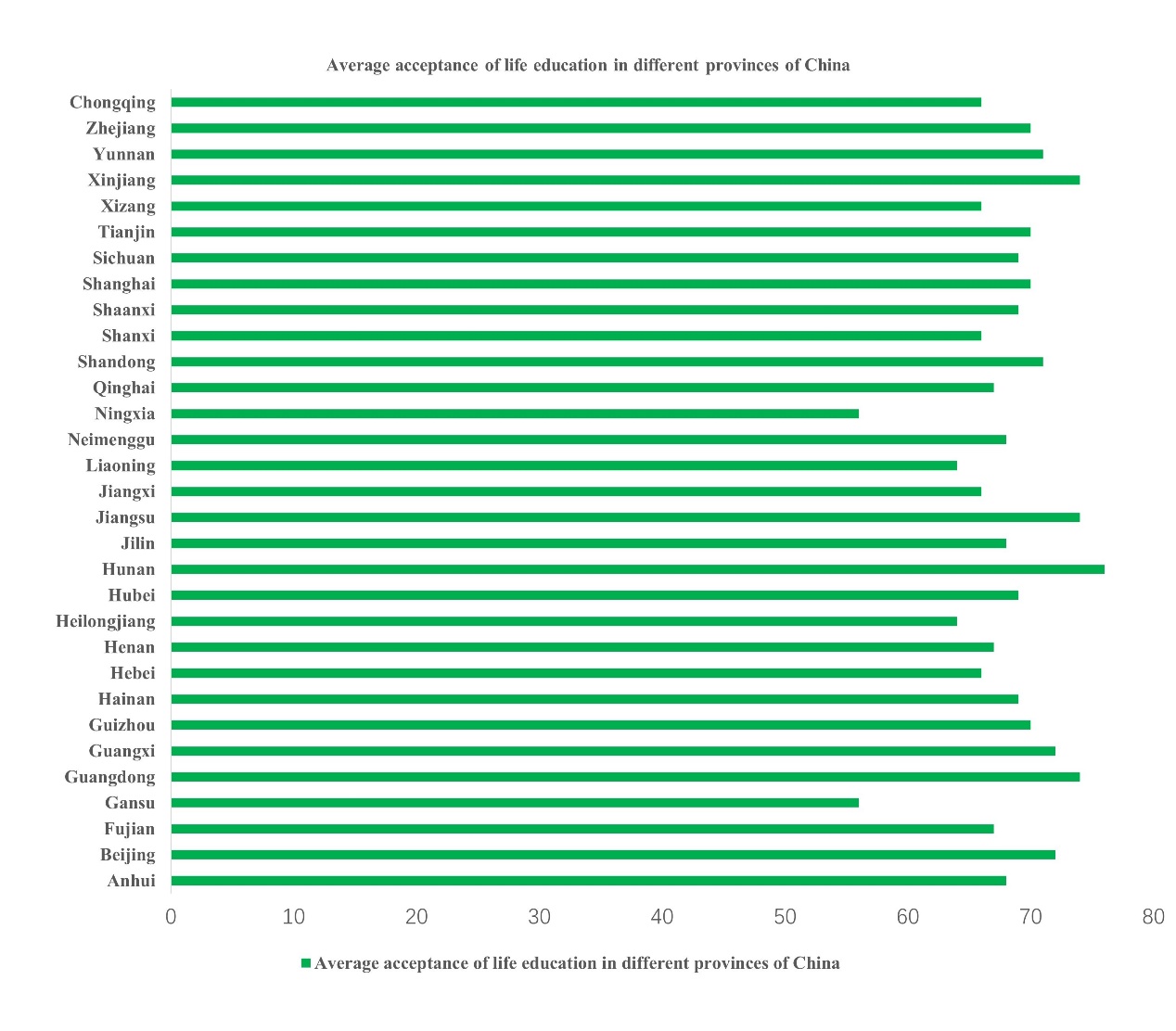


**Table S1. Measurement instruments and scoring (items, response scale, total range, transformations, reliability) used in mainland China, June–August 2022.**

| Instrument | Abbreviation | Construct Measured | Description / Scoring | **Reliability** |
| --- | --- | --- | --- | --- |
| EuroQol 5-Dimension 5-Level | **EQ-5D-5L** | Health-related Quality of Life (HRQoL) | Mobility, Self-care, Usual activities, Pain/discomfort, and Anxiety/depression. | The Cronbach's α=0.857 |
| Perceived Social Support Scale | **PSSS** | A self-report measure of subjectively assessed social support | Family support, friend support, other support | The Cronbach's α=0.923 |
| Family Health Scale | **FHS-SF** | Family Health | Family/social/emotional health processes, family healthy lifestyle, family health resources, and external social support for the family | The Cronbach's α= 0.84 |
| Health Literacy Scale | **HLS-SF** | Health Literacy | Medical care, disease prevention, and health promotion | The Cronbach's α= 0.94 |
| Media Exposure Scale |  | To assess the extent to which individuals are exposed to various forms of media | Seeking information, providing information, self-seeking, social interaction, and relaxation/entertainment | The Cronbach's α=0.85 |
| The World Health Organization-Five Well-Being Index | **WHO-5** | Positive Health Self-Assessment Scale | Emotional experience, vitality level, and functional performance | The Cronbach's α=0.83 |

**Table S2. Descriptive statistics of life education acceptance (VAS 0–100) in mainland China: national cross-sectional survey, June–August 2022.**

| **Variate** | | **Number** | **Proportion** | ***M*** | ***P*_25_** | ***P*_75_** | ***Z*/*H*** | ***P*** |
| --- | --- | --- | --- | --- | --- | --- | --- | --- |
| Total |  | 21875 |  | 71.00 | 50.00 | 95.00 |  |  |
| Gender |  |  |  |  |  |  |  |  |
|  | Male | 10942 | 50.02% | 73.00 | 51.00 | 99.00 | -8.59 | <0.001 |
|  | Female | 10933 | 49.98% | 70.00 | 49.00 | 92.00 |  |  |
| Nation |  |  |  |  |  |  |  |  |
|  | Ethnic Han | 19934 | 91.13% | 71.00 | 50.00 | 95.00 | -1.46 | 0.143 |
|  | Ethnic minorities | 1941 | 8.87% | 70.00 | 49.00 | 96.00 |  |  |
| Religion |  |  |  |  |  |  |  |  |
|  | No | 21005 | 96.02% | 72.00 | 50.00 | 96.00 | -4.91 | <0.001 |
|  | Yes | 870 | 3.98% | 65.50 | 46.00 | 86.00 |  |  |
| Occupational status |  |  |  |  |  |  |  |  |
|  | Incumbency | 7583 | 34.67% | 70.00 | 50.00 | 92.00 | 562.22 | <0.001 |
|  | Student | 6566 | 30.02% | 80.00 | 55.00 | 100.00 |  |  |
|  | Retire | 2749 | 12.57% | 69.00 | 50.00 | 89.00 |  |  |
|  | No fixed occupation | 2608 | 11.92% | 66.00 | 46.00 | 86.00 |  |  |
|  | Free job | 242 | 1.11% | 62.50 | 44.75 | 87.25 |  |  |
|  | Unemployment | 2127 | 9.72% | 62.00 | 43.00 | 83.00 |  |  |
| Highest educational level |  |  |  |  |  |  |  |  |
|  | Junior high or below | 6960 | 31.82% | 66.00 | 47.00 | 86.00 | 418.51 | <0.001 |
|  | Senior high or specialty | 7687 | 35.14% | 71.00 | 50.00 | 96.00 |  |  |
|  | Undergraduate or above | 7228 | 33.04% | 79.00 | 55.00 | 100.00 |  |  |
| Medicine specialty |  |  |  |  |  |  |  |  |
|  | No | 21137 | 96.63% | 71.00 | 50.00 | 94.00 | -10.19 | <0.001 |
|  | Yes | 738 | 3.37% | 85.00 | 60.00 | 100.00 |  |  |
| Have pets |  |  |  |  |  |  |  |  |
|  | No | 17232 | 78.77% | 71.00 | 50.00 | 95.00 | -3.13 | 0.002 |
|  | Yes | 4643 | 21.23% | 73.00 | 51.00 | 97.00 |  |  |
| Chronic disease diagnosis |  |  |  |  |  |  |  |  |
|  | No | 16223 | 74.16% | 72.00 | 50.00 | 97.00 | -3.87 | <0.001 |
|  | Yes | 5652 | 25.84% | 70.00 | 49.00 | 92.00 |  |  |
| Cancer diagnosis |  |  |  |  |  |  |  |  |
|  | No | 21769 | 99.52% | 71.00 | 50.00 | 95.00 | -0.22 | 0.827 |
|  | Yes | 106 | 0.48% | 69.00 | 48.75 | 95.25 |  |  |
| Place of residence |  |  |  |  |  |  |  |  |
|  | Rural | 6720 | 30.72% | 67.00 | 48.00 | 88.00 | -12.48 | <0.001 |
|  | Urban | 15155 | 69.28% | 73.00 | 51.00 | 99.00 |  |  |
| Marital status |  |  |  |  |  |  |  |  |
|  | Unmarried | 8479 | 38.76% | 79.00 | 53.00 | 100.00 | 396.15 | <0.001 |
|  | Married | 12417 | 56.76% | 68.00 | 49.00 | 88.00 |  |  |
|  | Divorce | 404 | 1.85% | 69.00 | 49.00 | 93.75 |  |  |
|  | Bereaved spouse | 575 | 2.63% | 67.00 | 41.00 | 87.00 |  |  |
| Number of cousins |  |  |  |  |  |  |  |  |
|  | 0 | 5859 | 26.78% | 78.00 | 53.00 | 100.00 | 188.31 | <0.001 |
|  | 1 | 5499 | 25.14% | 72.00 | 51.00 | 98.00 |  |  |
|  | 2 | 4207 | 19.23% | 68.00 | 49.00 | 90.00 |  |  |
|  | ≥3 | 6310 | 28.85% | 68.00 | 47.00 | 90.00 |  |  |
| Number of properties |  |  |  |  |  |  |  |  |
|  | 0 | 2475 | 11.31% | 67.00 | 45.00 | 93.00 | 344.43 | <0.001 |
|  | 1 | 13487 | 61.65% | 69.00 | 50.00 | 90.00 |  |  |
|  | 2 | 4393 | 20.08% | 78.00 | 55.00 | 100.00 |  |  |
|  | ≥3 | 1520 | 6.95% | 83.00 | 58.25 | 100.00 |  |  |
| Have debt |  |  |  |  |  |  |  |  |
|  | No | 13737 | 62.80% | 70.00 | 50.00 | 93.00 | -5.15 | <0.001 |
|  | Yes | 8138 | 37.20% | 73.00 | 51.00 | 99.00 |  |  |
| Average monthly household income |  |  |  |  |  |  |  |  |
|  | ≤1000 | 1373 | 6.28% | 60.00 | 41.00 | 88.00 | 312.61 | <0.001 |
|  | 1001-4000 | 9517 | 43.51% | 68.00 | 49.00 | 90.00 |  |  |
|  | 4001-9000 | 7904 | 36.13% | 74.00 | 52.00 | 98.00 |  |  |
|  | 9001-15000 | 2168 | 9.91% | 79.00 | 56.00 | 100.00 |  |  |
|  | ≥15001 | 913 | 4.17% | 81.00 | 59.00 | 100.00 |  |  |
| Age |  |  |  |  |  |  |  |  |
|  | 12-17 | 2170 | 9.92% | 75.00 | 51.00 | 100.00 | 527.51 | <0.001 |
|  | 18-24 | 4382 | 20.03% | 81.00 | 58.00 | 100.00 |  |  |
|  | 25-44 | 6604 | 30.19% | 69.00 | 50.00 | 92.00 |  |  |
|  | 45-64 | 5934 | 27.13% | 68.00 | 49.00 | 89.00 |  |  |
|  | >=65 | 2785 | 12.73% | 66.00 | 48.00 | 85.00 |  |  |
| Region |  |  |  |  |  |  |  |  |
|  | Eastern China | 8083 | 36.95% | 75.00 | 52.00 | 100.00 | 84.62 | <0.001 |
|  | Central China | 6905 | 31.57% | 70.00 | 50.00 | 95.00 |  |  |
|  | Western China | 6887 | 31.48% | 69.00 | 49.00 | 90.00 |  |  |
| Depression |  |  |  |  |  |  |  |  |
|  | No depression | 9295 | 42.49% | 75.00 | 52.00 | 98.00 | 161.57 | <0.001 |
|  | Mild depression | 7621 | 34.84% | 70.00 | 50.00 | 92.00 |  |  |
|  | Moderate depression | 3024 | 13.82% | 65.00 | 45.00 | 88.00 |  |  |
|  | Moderate to severe depression | 1418 | 6.48% | 68.00 | 47.00 | 95.25 |  |  |
|  | Severe depression | 517 | 2.36% | 82.00 | 54.00 | 100.00 |  |  |
| Anxiety |  |  |  |  |  |  |  |  |
|  | No anxiety | 11657 | 53.29% | 73.00 | 51.00 | 98.00 | 140.33 | <0.001 |
|  | Mild anxiety | 7137 | 32.63% | 69.00 | 49.00 | 90.00 |  |  |
|  | Moderate anxiety | 1783 | 8.15% | 64.00 | 44.00 | 88.00 |  |  |
|  | Moderate to severe anxiety | 957 | 4.37% | 70.00 | 50.00 | 99.00 |  |  |
|  | Severe anxiety | 341 | 1.56% | 86.00 | 50.00 | 100.00 |  |  |
| WHO-5 |  |  |  |  |  |  |  |  |
|  | poor physical and mental health status | 8662 | 39.60% | 67.00 | 47.00 | 91.00 | -12.61 | <0.001 |
|  | good physical and mental health status | 13213 | 60.40% | 74.00 | 52.00 | 98.00 |  |  |

**Table S3. Multicollinearity diagnostics (tolerance, VIF) for covariates in the multivariable model (VAS 0–100), mainland China, June–August 2022.**

| Variate | Tolerance | VIF |
| --- | --- | --- |
| Gender | 0.99 | 1.01 |
| Religion | 0.971 | 1.03 |
| Medicine specialty | 0.957 | 1.045 |
| Place of residence | 0.873 | 1.146 |
| Have debt | 0.946 | 1.057 |
| Highest educational level | 0.76 | 1.315 |
| Age | 0.737 | 1.357 |
| Region | 0.987 | 1.013 |
| Chronic disease diagnosis | 0.809 | 1.236 |
| PSSS | 0.619 | 1.614 |
| FHS-SF | 0.613 | 1.63 |
| HLS-SF | 0.644 | 1.553 |
| EQ-5D-5L | 0.89 | 1.124 |
| Media use | 0.634 | 1.578 |
