# Supplementary material for: Factors Affecting Acceptance of Life Education in Mainland China: National Cross-Sectional Study
Source: JMIR Public Health Surveill. 2026 Apr 21;12:e78844. doi: 10.2196/78844 (PMC13100709; doi:10.2196/78844)
Supplement: Checklist 1 [file publichealth-v12-e78844-s002.docx]

STROBE Statement—checklist of items that should be included in reports of observational studies

|  | Item No. | Recommendation | Page  No. | Relevant text from manuscript |
| --- | --- | --- | --- | --- |
| **Title and abstract** | 1 | (*a*) Indicate the study’s design with a commonly used term in the title or the abstract | 1 | The title states 'a national cross-sectional study,' and the abstract clearly points out 'This national cross-sectional study'. |
|  |  | (*b*) Provide in the abstract an informative and balanced summary of what was done and what was found | 1 | The abstract includes background, objectives, methods, results, and conclusions, with a clear structure and complete information. |
| Introduction | | | |  |
| Background/rationale | 2 | Explain the scientific background and rationale for the investigation being reported | 2-3 | Introduces the definition of life education, the development status at home and abroad, the taboos surrounding death in the context of Chinese culture, and the necessity of the research. |
| Objectives | 3 | State specific objectives, including any prespecified hypotheses | 3 | “the present study will examine public acceptance of life education and identify factors associated with receptivity and willingness to engage” |
| Methods | | | |  |
| Study design | 4 | Present key elements of study design early in the paper | 3-10 | **Method**  **Research instrument** has been described as a cross-sectional study, and the methods section is also described in detail. |
| Setting | 5 | Describe the setting, locations, and relevant dates, including periods of recruitment, exposure, follow-up, and data collection | 4 | The data collection period was from June 20 to August 31, 2022, covering 23 provinces, 5 autonomous regions, and 4 municipalities directly under the central government. |
| Participants | 6 | (*a*) *Cohort study*—Give the eligibility criteria, and the sources and methods of selection of participants. Describe methods of follow-up  *Case-control study*—Give the eligibility criteria, and the sources and methods of case ascertainment and control selection. Give the rationale for the choice of cases and controls  *Cross-sectional study*—Give the eligibility criteria, and the sources and methods of selection of participants | 4 | Clearly lists six inclusion and exclusion criteria, and describes the methods of stratified sampling and quota sampling. |
|  |  | (*b*) *Cohort study*—For matched studies, give matching criteria and number of exposed and unexposed  *Case-control study*—For matched studies, give matching criteria and the number of controls per case | **Not applicable** |  |
| Variables | 7 | Clearly define all outcomes, exposures, predictors, potential confounders, and effect modifiers. Give diagnostic criteria, if applicable | 4-9 | It lists sociological information such as gender, age, major, region, and educational background, as well as scales for family health and media exposure. |
| Data sources/ measurement | 8* | For each variable of interest, give sources of data and details of methods of assessment (measurement). Describe comparability of assessment methods if there is more than one group | 3-4 | Provides a detailed description of the questionnaire structure as well as the PSSS-3, FHS-SF, HLS-SF, EQ-5D-5L, Media Use Scale, and WHO-5 measurement tools |
| Bias | 9 | Describe any efforts to address potential sources of bias | 4-5 | Methods such as stratified sampling, quota control, standardized training, and face-to-face surveys are used to reduce bias. |
| Study size | 10 | Explain how the study size was arrived at | 4 | Calculate the minimum sample size using formulas and explain quota control based on census data. |

Continued on next page

| Quantitative variables | 11 | Explain how quantitative variables were handled in the analyses. If applicable, describe which groupings were chosen and why | 5-9，  Table 2-7 | Such as age grouping, education level grouping, and continuous variable treatment of scale scores |
| --- | --- | --- | --- | --- |
| Statistical methods | 12 | (*a*) Describe all statistical methods, including those used to control for confounding | 4 | Use a generalized linear model to adjust for multiple confounding factors |
|  |  | (*b*) Describe any methods used to examine subgroups and interactions | 4,  Table 2-7, | Subgroup analysis was conducted based on medical background and age group |
|  |  | (*c*) Explain how missing data were addressed | 4 | Missing data were not present for the variables included in the analyses; therefore, no imputation was performed. |
|  |  | (*d*) *Cohort study*—If applicable, explain how loss to follow-up was addressed  *Case-control study*—If applicable, explain how matching of cases and controls was addressed  *Cross-sectional study*—If applicable, describe analytical methods taking account of sampling strategy | 4 | Use quota sampling and take sample representativeness into account in the analysis |
|  |  | (*e*) Describe any sensitivity analyses | 4 | In addition, sensitivity analyses were conducted for selected measurement instruments to evaluate the robustness of results to alternative scoring and operationalization of key scales; |
| Results | | | | |
| Participants | 13* | (a) Report numbers of individuals at each stage of study—eg numbers potentially eligible, examined for eligibility, confirmed eligible, included in the study, completing follow-up, and analysed | 5, 10  Figure 1 | Figure 1 clearly shows the sample selection process. |
|  |  | (b) Give reasons for non-participation at each stage | 4 | The exclusion criteria specify the situations in which participation is not allowed. |
|  |  | (c) Consider use of a flow diagram | Figure1 | Participant flow for a national cross-sectional survey of community residents in mainland China, June–August 2022. |
| Descriptive data | 14* | (a) Give characteristics of study participants (eg demographic, clinical, social) and information on exposures and potential confounders | 5,10,  Table 1&S1 | The supplementary materials provide complete demographic characteristics. |
|  |  | (b) Indicate number of participants with missing data for each variable of interest | **Not applicable** | All unqualified samples have been removed during the survey stage. |
|  |  | (c) *Cohort study*—Summarise follow-up time (eg, average and total amount) |  |  |
| Outcome data | 15* | *Cohort study*—Report numbers of outcome events or summary measures over time |  |  |
|  |  | *Case-control study—*Report numbers in each exposure category, or summary measures of exposure |  |  |
|  |  | *Cross-sectional study—*Report numbers of outcome events or summary measures | 10, Table 1 | Provided the median, quartiles, and regression analysis results of VAS scores |
| Main results | 16 | (*a*) Give unadjusted estimates and, if applicable, confounder-adjusted estimates and their precision (eg, 95% confidence interval). Make clear which confounders were adjusted for and why they were included | 10, Table 1 | Provides the adjusted β values and 95% CI |
|  |  | (*b*) Report category boundaries when continuous variables were categorized | 5-9 | Both age groups and education groups have clear boundaries |
|  |  | (*c*) If relevant, consider translating estimates of relative risk into absolute risk for a meaningful time period | **Not applicable** |  |

Continued on next page

| Other analyses | 17 | Report other analyses done—eg analyses of subgroups and interactions, and sensitivity analyses | 5-9,  Table2-7 | Provided subgroup analyses by medical specialty and age group |
| --- | --- | --- | --- | --- |
| Discussion | | | | |
| Key results | 18 | Summarise key results with reference to study objectives | 10-12，  Figure 2-3 | Summarized the influencing factors such as gender, age, education, and media use |
| Limitations | 19 | Discuss limitations of the study, taking into account sources of potential bias or imprecision. Discuss both direction and magnitude of any potential bias | 17 | Discussed cross-sectional design, limitations of causal inference, residual confounding, etc. |
| Interpretation | 20 | Give a cautious overall interpretation of results considering objectives, limitations, multiplicity of analyses, results from similar studies, and other relevant evidence | 13-17 | Explain by integrating cultural background, policy recommendations, and previous research |
| Generalisability | 21 | Discuss the generalisability (external validity) of the study results | 17 | The sample covers the entire country and is somewhat representative, but it still needs to be promoted with caution. |
| Other information | |  | | |
| Funding | 22 | Give the source of funding and the role of the funders for the present study and, if applicable, for the original study on which the present article is based | 17 | **Funding**  This study was supported by the CAMS Innovation Fund for Medical Sciences (CIFMS) (Grant No. 2023-I2M-2-001). The funder had no involvement in the study design, data collection, analysis, interpretation, or the writing of the manuscript. |

*Give information separately for cases and controls in case-control studies and, if applicable, for exposed and unexposed groups in cohort and cross-sectional studies.

**Note:** An Explanation and Elaboration article discusses each checklist item and gives methodological background and published examples of transparent reporting. The STROBE checklist is best used in conjunction with this article (freely available on the Web sites of PLoS Medicine at http://www.plosmedicine.org/, Annals of Internal Medicine at http://www.annals.org/, and Epidemiology at http://www.epidem.com/). Information on the STROBE Initiative is available at www.strobe-statement.org.
